# Unsuccessful Presentations for Involuntary Admission to an Irish Approved Centre During 2019 and 2021

**DOI:** 10.1192/bjo.2024.602

**Published:** 2024-08-01

**Authors:** Cathal McCaffrey, Mark Daubaras, Shahrizal Ab Karim, Alyson Lee

**Affiliations:** Lakeview Unit, Naas, Ireland

## Abstract

**Aims:**

Ireland's *Mental Health Act 2001* (MHA) outlines the procedure and criteria for referring patients for involuntary admission. After consultant psychiatrist examination, if appropriate, a referred individual is admitted involuntarily under an admission order (AO). Involuntary admission is only appropriate if the person meets criteria for a “*Mental Disorder”*. It's unlawful to detain a person solely because they suffer from personality disorder, are socially deviant, or are addicted to drugs/intoxicants. AOs aren't completed if these criteria aren't met, referral forms are incorrect, or individuals agree to voluntary admission. We aimed to determine (1) the rate of unsuccessful referrals for involuntary admission to an Irish approved centre (Lakeview Unit) during 2019 and 2021, (2) the reasons AOs weren't completed, (3) the source of unsuccessful referrals and (4) the time such referrals were made.

**Methods:**

Unsuccessful referrals for involuntary admission during 2019 and 2021 were identified. Data were collected retrospectively by chart review. We identified the documented reason AOs were not completed, and the time and source of these unsuccessful referrals. In March 2021, authors delivered teaching on appropriate use of the MHA to local Police (Gardaí) and out-of-hours General Practitioners (GPs).

**Results:**

Of 78 referrals for involuntary admission in 2019 and 115 in 2021, 19% and 44% respectively were unsuccessful (AO not signed). In 60% of unsuccessful referrals in 2019, the person presented with no mental disorder meeting criteria for involuntary detention. The same figure in 2021 was 27%. Individuals presented with substance misuse disorder in 73% and 27% of unsuccessful referrals in 2019 and 2021 respectively. In 2019, 93% of unsuccessful referrals came from Gardaí, 100% came from medical practitioners other than the patient's own GP and 73% came outside of normal working hours.

**Conclusion:**

Unsuccessful referrals for involuntary admission are not uncommon. Audit cycle 1 highlighted that those subject to unsuccessful referrals frequently present out-of-hours, with substance misuse disorder and/or no mental disorder meeting criteria for involuntary admission. In 2021 a smaller proportion of unsuccessful referrals presented with no mental disorder and substance misuse disorder. This suggests that Gardaí/GP education offered benefit. We observed an increase in both the overall number of referrals and the percentage of unsuccessful referrals in 2021. This possibly reflects an impact of the COVID-19 pandemic. There is a need for ongoing education, discussion and feedback with Gardaí and GPs on the process of making referrals for involuntary admission. A limitation is that separate registrars completed each audit cycle.

